# Diseases of the Upper Respiratory Tract

**Published:** 1901-06

**Authors:** 


**Diseases of the Upper Respiratory Tract.** By P. WATSON WILLIAMS, M.D. Lond. Fourth Edition. Pp. xxiv., 436. Bristol: J. Wright & Co. 1901.

The new edition of this work is not only twice the size of the previous edition, but it contains so many improvements both in the text and in illustrations that it may fairly be described as a new work. Perhaps the principal feature of the book is the presence of 37 large-sized and admirably-executed plates. Some are coloured drawings and others are stereoscopic reproductions of anatomical preparations illustrating the anatomy of the various sinuses of the skull, the nasal fossæ, the pharynx, the distribution of nerves and vessels to the neck, the middle ear, &c. They are drawn from various sources, but some of the most striking are from the museum of University College, Bristol, while others are from the Royal College of Surgeons, the Westminster Hospital Museum, &c. Each copy of the book is accompanied by a small and simple stereoscope, which, with a little practice, enables the student to obtain an admirable stereoscopic view of the clinical anatomy of the nose, pharynx, larynx, sinuses, &c., familiarity with which is essential for a thorough understanding of the diseases of these parts. Diseases of the ear are not included in the book, but there is an excellent chapter on examination of the ear, a knowledge of which is essential to the proper understanding of diseases of the pharynx and nose; on the other hand, diseases of the nasal cavities, pharynx, naso-pharynx (which the author prefers to call rhino-pharynx), and the accessory sinuses are admirably and fully treated. Adenoids are amply described. The author favours the use of Gottstein's curette as modified by Delstanche, and he places the patient in the recumbent position with the head hanging over the end of the table.

For the removal of soft gelatinous polypi from the nose we are glad to find that Dr. Watson Williams favours the use of avulsion. It has been too much the fashion of late for specialists altogether to decry the use of forceps for this purpose and to allow no instruments but snares or cutting scissors. No doubt in unskilful hands much damage may be done by forceps in this region, but if carefully used, always with a good reflected light, we are sure that for many cases at least no instrument is so handy or useful as the forceps. We are surprised at the author's statement that "Sarcoma is rarely primary in the nose, while carcinoma is extremely rare." We have seen so many cases of primary malignant disease in this region, that such cases can hardly be rare; perhaps they are most frequently seen in the practice of the general surgeon.

The chapter on diphtheria is one of the best and fullest in the book. The author believes in a membranous croup which is not diphtheria, but which has a high mortality. He points out very clearly the close connection between fibrinous rhinitis and diphtheria, and gives an excellent description of the different forms of bacilli met with in diphtheritic affections. We note the omission of any statement as to the period of incubation of diphtheria; this is a matter which is often referred to by practitioners, and some information should be given, even if it cannot be dogmatic.

Dr. Watson Williams still believes in the old-fashioned "steam-bed" in the treatment of membranous croup, and he favours intubation rather than tracheotomy in this disease. A good description of the operation of intubation is given, with illustrations of the instruments, but no account of the operation of tracheotomy can be found; this surely is an omission. The various forms of paralysis of the vocal cords are admirably described, and full acknowledgment is made of the work of Felix Semon in this department. Perhaps sufficient prominence is hardly given to the functional paralysis known as "hysterical aphonia." On looking in the otherwise copious index for this disease we failed to find it, and the tyro might easily fail to notice it under the heading of "Bilateral Functional Adductor Paralysis."

Diseases of the nasal accessory sinuses have come much into notice during the last few years. Dr. Watson Williams considers that the large majority of cases of sinusitis have succeeded to attacks of influenza, and it is only since the occurrence of the influenza epidemics commencing in the year 1889 that inflammatory diseases of the accessory sinuses have become common affections.

The stereoscopic plates at the end of the book are particularly valuable in illustrating the relations of the various sinuses and their channels of communication with the nose. The student of these parts will owe a debt of gratitude to Dr. Watson Williams for these admirable pictures, as well as for the lucid descriptions which are appended. We believe it is the first time that the stereoscope has been introduced into a book, and the stereoscopic plates will be quite a revelation to those who have not previously seen such pictures of anatomical subjects by stereoscopic aid.

The work is published in two forms, either as one complete volume, or in two parts—Part I. being the text complete, with over 200 illustrations; while Part II. is the atlas of plates, with the stereoscope. Doubtless some students will be glad of the alternative which enables them to buy an extremely cheap, handy, and complete text-book, to which Part II. may be added later if desired, or *vice versâ*, although we feel sure the possessors of either part will take the earliest opportunity of completing the volume.

The book as a whole will fill a much-needed want, and will take its place as one of the most complete works on the subject in the English language.

We can but congratulate the author and the publishers on the form of the book and its moderate price.

